# Use of green fluorescent nano-sensors for the determination of furosemide in biological samples and pharmaceutical preparations

**DOI:** 10.1186/s13065-023-00937-y

**Published:** 2023-03-24

**Authors:** Mona H. Abo Zaid, Nahed El-Enany, Aziza E. Mostafa, Ghada M. Hadad, Fathalla Belal

**Affiliations:** 1 Pharmaceutical Chemistry Department, Faculty of Pharmacy, Delta University for Science and Technology, Gamasa, 35712 Egypt; 2 Pharmaceutical Analytical Chemistry Department, Faculty of Pharmacy, Suez Canal University, Ismailia, 41522 Egypt; 3 Pharmaceutical Analytical Chemistry Department, Faculty of Pharmacy, Mansoura University, Mansoura, 35516 Egypt; 4 Pharmaceutical Chemistry Department, Faculty of Pharmacy, New Mansoura University, New Mansoura, 7723730 Egypt

**Keywords:** Sucrose, Urea, Quantum dots, Furosemide, Human Plasma

## Abstract

**Background:**

Carbon quantum dots (CQDs) are new class of carbon nanoparticles. Recently, they have been widely used as fluorescent probes due to their easy accessibility, optical properties and chemical inertness. Many available precursors are used in the synthesis of carbon quantum dots. The electrical and optical properties of CQDs could be enhanced by doping hetero atoms such as nitrogen or sulfur into their structure.

**Objective:**

The current work presents the synthesis and characterization of water-soluble nitrogen doped carbon quantum dots (N-CQDs) and their use as fluorescent nano-sensors for the spectrofluorimetric determination of furosemide in its pharmaceutical preparations and spiked human plasma.

**Methods:**

A domestic microwave was used to prepare the N-CQDs by heating a solution of sucrose and urea till complete charring (about ten minutes). The produced N-CQDs exhibit a strong emission band at 376 nm after excitation at 216 nm. Furosemide caused a quantitative quenching in the fluorescence intensity of the produced N-CQDs.

**Results:**

The proposed method was validated according to ICH Guidelines. The method was found to be linear over the range of 0.1–1.0 µg/mL with LOQ of 0.087 µg/ml.

**Conclusion:**

Ecofriendly nano fluorescent sensors (N-CQDs) were successfully synthesized. The size of N-CQDs was distributed in the range of 6.63 nm to 10.23 nm with an average of 8.2 nm. The produced N-CQDs were used as fluorescent probes for the estimation of furosemide in its pharmaceutical preparations as well as spiked human plasma samples.

## Introduction

**Fig. 1:**
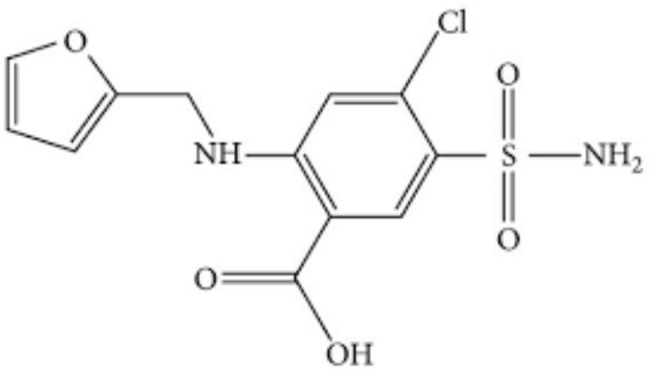
Chemical structure of Furosemide

Furosemide (FRS) is frequently used to treat edema associated with heart failure and hepatic or renal problems. It is a potent loop diuretic. It may be effective in patients who are not responding to thiazide diuretics [1]. It is given orally and may be given intravenously or intramuscularly as its sodium salt. Most side effects of the drug happen with high doses, such as electrolyte and fluid imbalance (hyponatremia and hypokalemia) mainly after prolonged use or large doses. The common symptoms of electrolyte imbalance are hypotension, headache, dry mouth, thirst, muscle cramps, restlessness, weakness, drowsiness, lethargy, oliguria, gastrointestinal disturbances and cardiac arrhythmias. Furosemide is fairly rapidly absorbed from GIT; bioavailability of the drug is about 60 to 70% but absorption is variable. The reported half-life time is up to about two hours although it is prolonged in patients with hepatic and renal impairment. It is mainly excreted unchanged in the urine. Non-renal elimination is increased in renal impairment. Furosemide is bound to plasma albumin up to 99% ^1,2^. Chemically, furosemide is: 4-Chloro-2-(furan-2-ylmethylamino)-5-sulfamoylbenzoic acid [2, 3] (Fig. 1). The reported methods for analysis of FRS in dosage forms and biological fluids include spectrophotometry [4–6], LC-MS [7], HPTLC [8], RP-HPLC [9–11] and spectrofluorimetry [12, 13]. Most of these techniques require multi-steps procedures and expensive instrumentation [7–10] for its analysis. Developing a sensitive spectrofluorimetric method for estimation of FRS in pharmaceutical preparations and human plasma samples without prior derivatization was the aim of this work. N-Doped CQDs were used for this purpose and the results were promising. The proposed method depends on synthesis of N-CQDs by domestic microwave-assisted method using mixture of sucrose and urea in water. Water serves as a highly green solvent. The method is based on formation of N-CQDs, which could be used as fluorescent nano-sensors for estimation of FRS. The drug caused quantitative quenching of the fluorescent N-CQDs. The major advantages of the proposed method are the high speed of production of N-CQDs in only about thirty minutes with high quantum yield (0.57) and the ability to detect down to nano-gram concentrations of the drug. Carbon quantum dots (CQDs) are a new generation of fluorescent nano particles characterized by being low in cost, photo-stable, safe or not toxic, very good biocompatible and highly water soluble [14–17]. In addition, the electrical and optical properties of CQDs could be boosted by doping hetero atoms such as nitrogen or sulfur into the structure of CQDs [18, 19].

## Experimental

### Materials and reagents

- Methanol (HPLC grade) was obtained from Fisher (Belgium).
- Orthophosphoric acid 85% and boric acid: (Sigma-Aldrich, Switzerland).
- Glacial acetic acid 99% :(Alfa Chemical Group, Cairo, Egypt).
- Britton Robinson Buffer (BRB)was prepared by mixing each of 0.04 M boric acid, 0.04 M phosphoric acid and 0.04 M acetic acid, then the pH was adjusted using 0.2 M sodium hydroxide over the range from 2.1 to 12.
- Furosemide was kindly supplied by NODCAR, Cairo, Egypt, its purity was 99.9%.
- Lasix® tablets: 40 mg/tablet (Batch No. BEG009) and Lasix® ampoules 20 mg/2mL (Batch No. BEG010). Products of Sanofi Pharmaceutical Company, Cairo, Egypt. Both were purchased from local Pharmacy.
- Deionized water was used throughout the work.
- Human plasma samples were kindly supplied by Mansoura University Hospital, Mansoura, Egypt. Samples were kept frozen at − 80 °C until use after gentle thawing.

### Instrumentation

- Shimadzu RF-6000 series Fluorescence Spectrophotometer with 150 W Xenon lamp was used for Fluorescence measurements. High sensitivity mode was used throughout the work.
- Shimadzu UV-Visible Spectrophotometer, Model 1900i (Japan), was used for the spectrophotometric measurements.
- The FT-IR spectrum was recorded using Thermo Fisher Scientific Nicolet - iS10 FT-IR Spectrometer (168 Third Avenue Waltham, MA, USA).
- The produced N-CQDs were examined for their morphology using JEM-2100 high resolution transmission electron microscope (HRTEM) (JEOL, Tokyo). The instrument was operated at 200 KV.
- Vortex mixer, ZX3 (Velp. Scientific) Italy.
- Ultrasonic bath, Elma Sonic S 100(H), Germany.
- Centrifuge, model sigma 2-16P, Germany.
- Jenway3510 pH-Meter, UK.
- Domestic LG microwave Model No: MH7043BARS (Power Input 230 V AC /50Hz, output of 900 W and frequency of 2,450 MHz.

### Standard solutions

Stock solution (1000.0 µg/mL) of FRS was prepared by weighing out 10.0 mg of FRS, placing it in 10 mL volumetric flask and dissolve it in methanol. Different concentrations were prepared by diluting the stock solution as appropriate to obtain working solutions.

### Preparation of N-CQDs

The fluorescent N-CQDs were synthesized as previously reported [19] using 15 g of sucrose and 3 g of urea dissolved in 30 mL of deionized water then heated in microwave for 10 min to ensure complete charring. The resulting N-CQDs were left to cool, diluted with water and centrifuged at 6000 rpm for 10 min. Then, 0.22 μm Syringe filters were used to remove the suspended impurities. Finally, the volume of N-CQDs was adjusted with deionized water to final volume of 100 mL to get stock solution.

### General Procedure

#### For raw material

Aliquots of 400 µL of N-CQDs were transferred into a series of 10 mL measuring flasks. Various volumes from working standard solution (10.0 µg/mL) of FRS were added to each flask. 1 mL of BRB of pH 6.0 was also added. The solutions were completed to the mark with deionized water. The values of fluorescence quenching (ΔF) were estimated at 376 nm using an excitation wavelength of 216 nm). The calibration curve was plotted between ΔF versus final drug concentration in (µg/mL). Consequently, the linear regression equation was derived.

### Pharmaceutical preparations

#### Lasix® tablets

Ten of Lasix® tablets were weighed individually, transferred to a mortar and powdered. A weight equivalent to 10.0 mg of FRS was quantitatively transferred into a small flask followed by 5 ml of methanol. The flask was sonicated for 10 min, then filtered into 10 ml measuring flask. Methanol was used to adjust the volume to give a solution of (1000.0 µg/mL). Methanol was used to dilute 0.1mL aliquot to give solution of 10.0 µg/mL in a 10.0 volumetric flask. The described procedure above **(For Raw Material)** was then applied. % Recoveries were calculated adopting the corresponding regression equation.

#### Lasix® Ampoules

The contents of ten ampoules of Lasix® were transferred to a beaker. 1.0 mL from the mixed solutions was transferred into 10 mL volumetric flask followed by 5 ml of methanol. The same solvent was used to adjust the volume to give a solution of 1000.0 µg/mL. Then 0.1mL aliquots were diluted to 10.0 mL with methanol to give solution of 10.0 µg/mL. The described procedure under section **(For Raw Material)** was adopted. Then the regression equation was used to calculate % recoveries.

#### Spiked human plasma

Aliquots of FRS standard working solution were quantitatively transferred into a series of 15 mL centrifuge tubes each containing 1 mL of human plasma then vortexed for 30 s. Acetonitrile up to 5 mL was added to each tube. Centrifugation at 3600 rpm was carried out for 30 min. Aliquots of 1.0 ml of the produced supernatant were transferred into 10.0 mL volumetric flasks, completed to the mark, and the described procedure **(For Raw Material)** was applied. The percentage recoveries were calculated adopting the regression equation.

## Results and discussion

The present study reports a rapid, simple, cost-effective and sensitive spectrofluorometric method for estimation of FRS. The method of synthesis of N-CQDs is green and rapid using simple procedure and available starting materials compared to other reported methods [18, 19]. The method of synthesis takes only 30 min to produce highly fluorescent probe which could be used for its determination.

### Characterization of N-CQDs

**Fig. 2:**
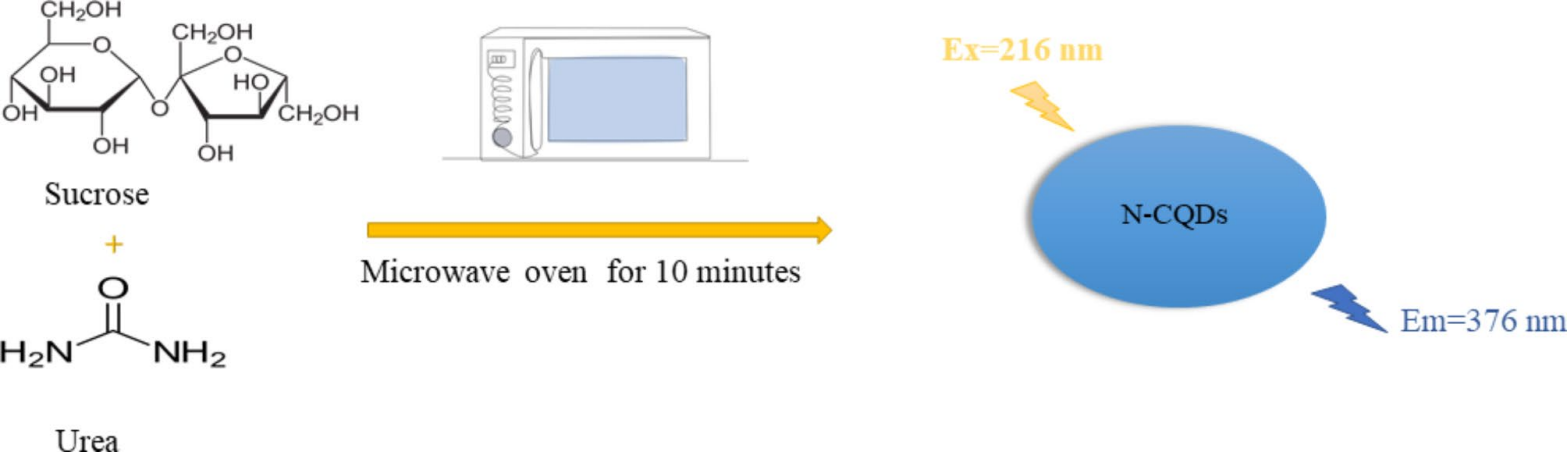
General procedure for synthesis of N-CQDs.

**Fig. 3:**
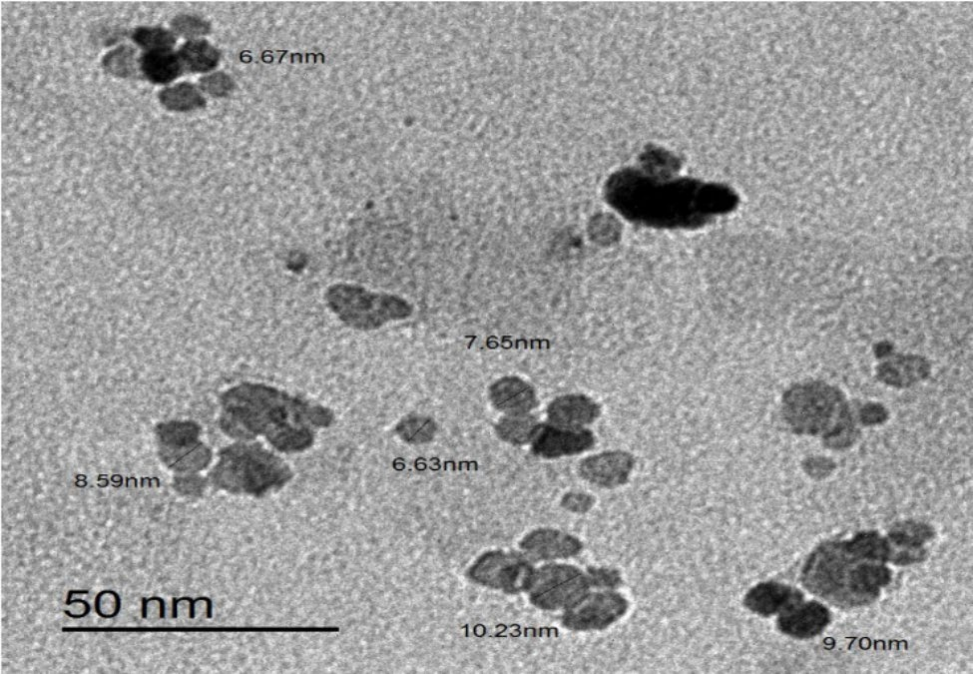
The typical HRTEM images of the N-CQDs.

**Fig. 4:**
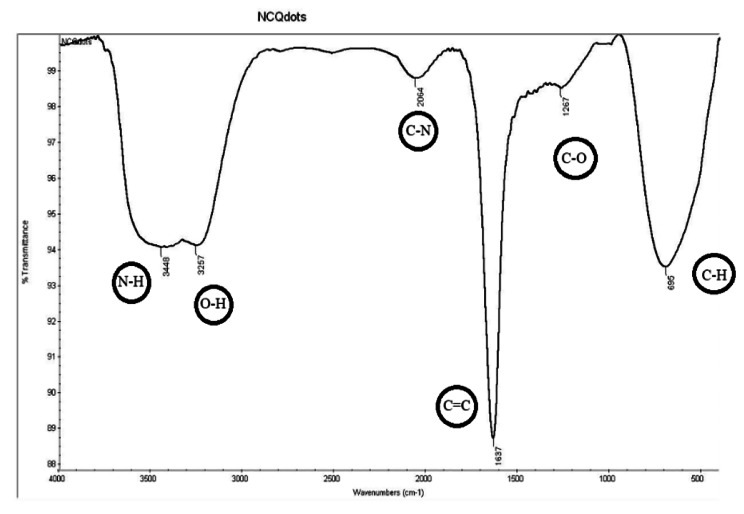
FT-IR spectra of N-CQDs.

**Fig. 5:**
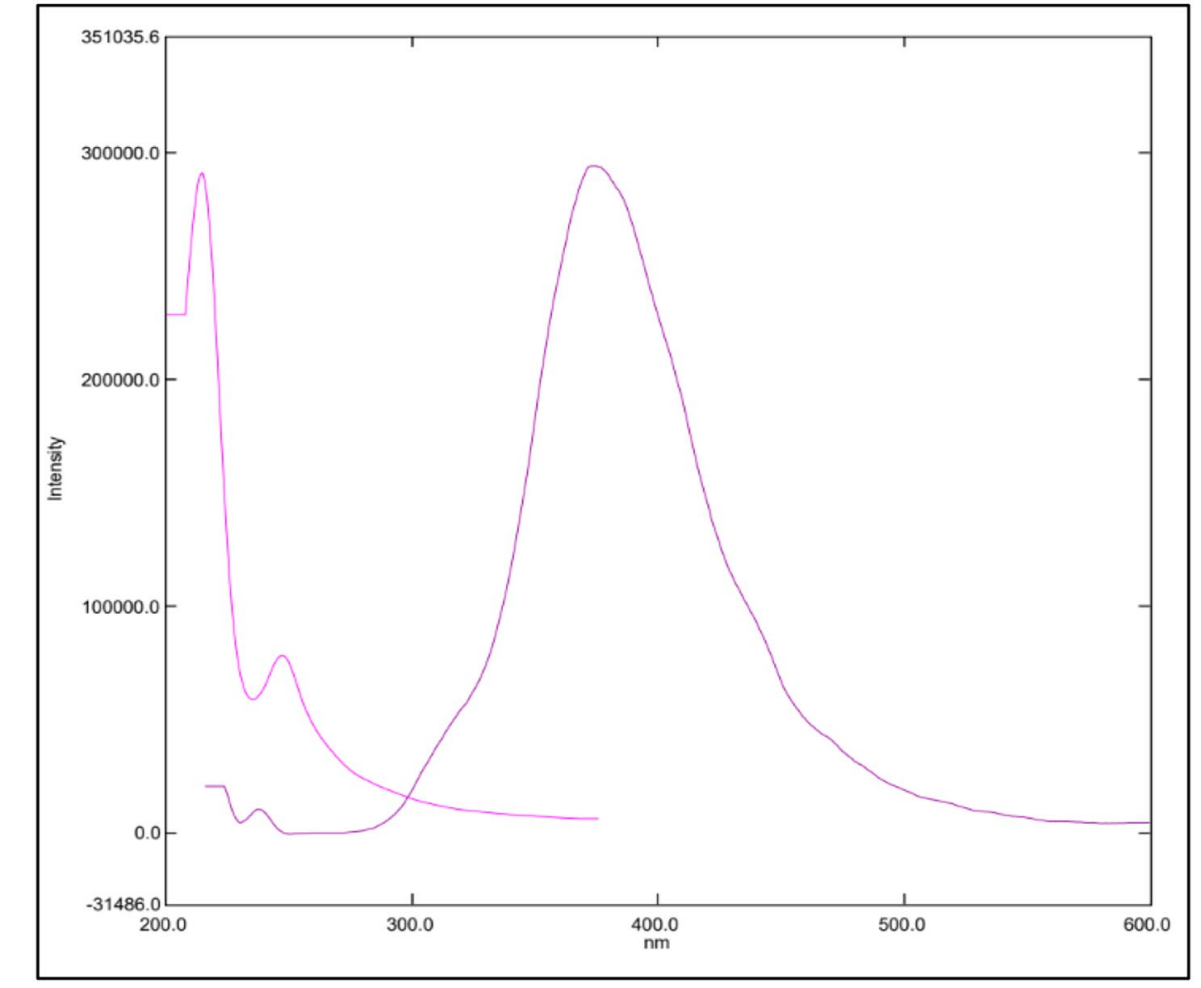
Fluorescence excitation and emission spectra of N-CQDs.

**Fig. 6:**
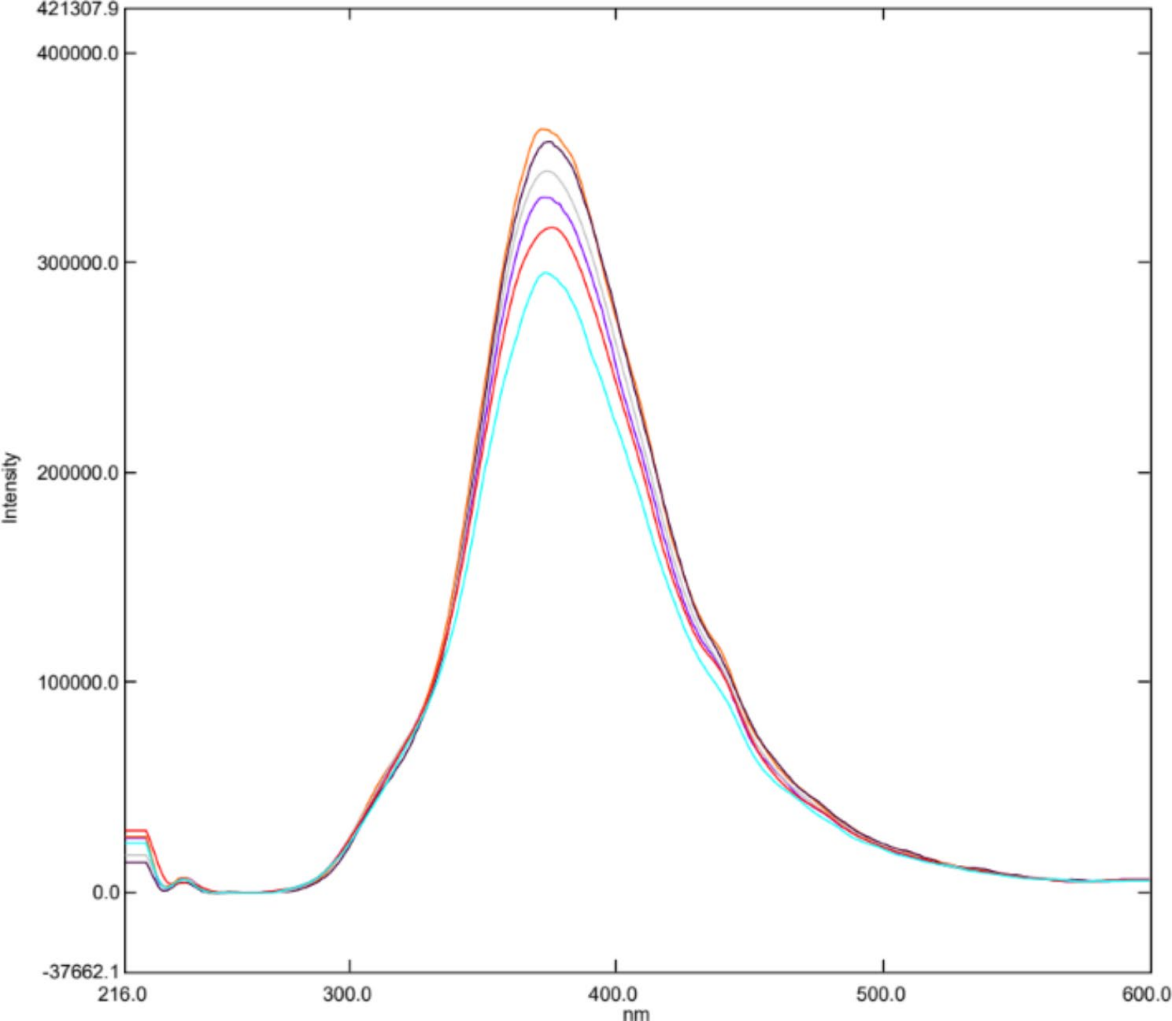
Fluorescence emission spectra of the N-CQDs in aqueous solution upon addition of various concentrations of FRS (from top to bottom: 0 µg/mL, 0.1 µg/mL, 0.3 µg/mL, 0.5 µg/mL, 0.7 µg/mL, 1.0 µg/mL)

**Fig. 7:**
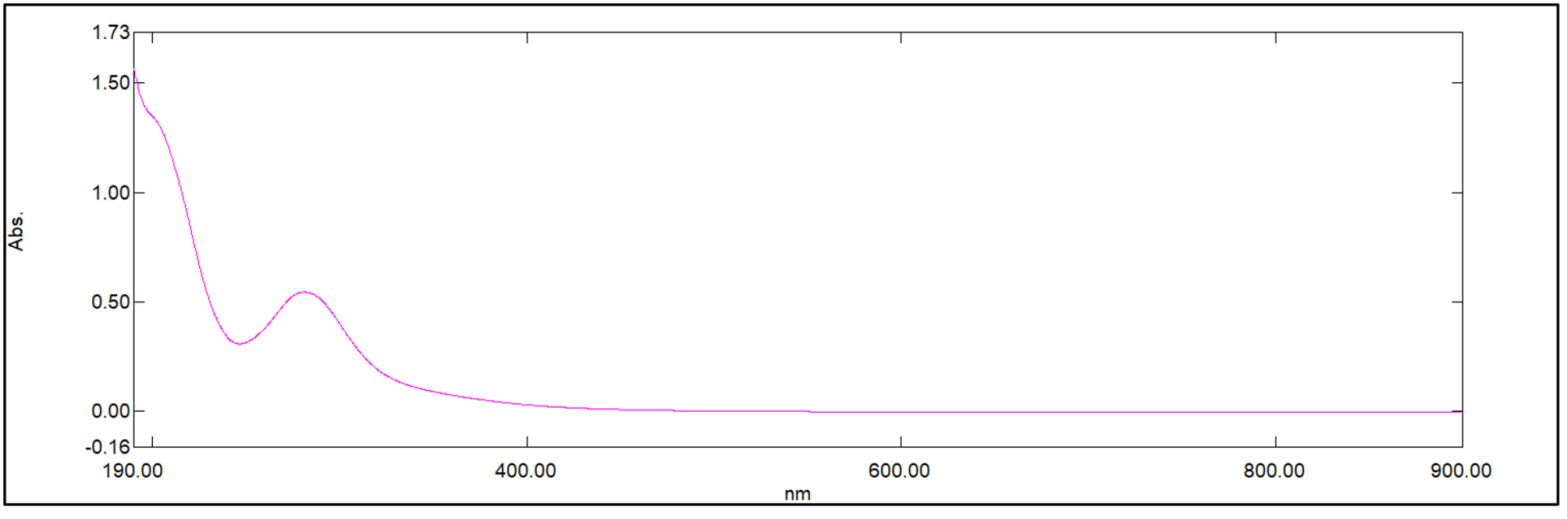
The UV–VIS absorption spectrum of N-CQDs.

Water-soluble N-CQDs were synthesized using sucrose as a carbon source and urea as a nitrogen source using microwave assisted method. An aqueous solution of orange color indicating the production of N-CQDs (Fig. 2). TEM image of N-CQDs is presented in (Fig. 3) showings spherical N-CQDs with diameters in the range of 6.63 nm to 10.23 with an average of 8.2 nm. (FT-IR) analysis was also carried out to identify N-CQDs functional groups (Fig. 4). The broad band in the range of 3600–3100 cm^− 1^ is characteristic for N-H and O-H groups, the peak at 1638 cm − 1indicates the presence of C = C/C = O. The vibrations at 1265 cm^− 1^ represents the stretching modes of C-O-C band [20]. The high fluorescence intensity of the produced N-CQDs is proved by the emission and excitation spectra of fluorescent N-CQDs in deionized water as presented in (Fig. 5). FRS has an obvious quenching effect on N-CQDs as presented in (Fig. 6). The UV-VIS absorption spectrum of the formed quantum dots is presented in (Fig. 7).

### Quantum Yield

In the present work, the N-CQDs are characterized by high quantum yield (0.57). Equation 1 was used to calculate their quantum yield by the single point method adopting the following formula [21]:

1 $${\Phi _{\rm{x}}} = {\rm{ }}{\Phi _{{\rm{st}}}} \times {\rm{ }}\left( {{{\rm{F}}_{\rm{x}}}/{{\rm{F}}_{{\rm{st}}}}} \right){\rm{ }} \times {\rm{ }}\left( {{{\rm{\eta }}_{\rm{x}}}/{{\rm{\eta }}_{{\rm{st}}}}} \right){\rm{ }} \times {\rm{ }}\left( {{{\rm{A}}_{{\rm{st}}}}/{{\rm{A}}_{\rm{x}}}} \right)$$

In which:

Φ refers to the quantum yield, (x) and (st) subscripts refer to the unknown sample and the standard solutions. F is the integrated measured intensity of emission, A is the absorbance and η indicates the refractive index of the solvent. In aqueous solutions, η_x_/η_st_ is equal to 1. Quinine sulfate in 0.1 M sulfuric acid was used as the standard fluorescent substance (QY: 0.54) [21].

### Fluorescence response mechanism of N-CQDs to FRS

The process of fluorescence quenching can be resolved into different types, dynamic, static quenching and inner filter effect. For better understanding of the mechanism of quenching, Stern Volmer’s Eq. (2) was used to investigate the fluorescence emission intensity of the N-CQDs FRS system [22].

2 $${{\rm{F}}_0}/{\rm{F }} = {\rm{ }}1{\rm{ }} + {\rm{ }}{{\rm{K}}_{\rm{q}}}{{\rm{\tau }}^{\rm{0}}}\left[ {\rm{Q}} \right]{\rm{ }} = {\rm{ }}1{\rm{ }} + {\rm{ }}{{\rm{K}}_{{\rm{sv}}}}\left[ {\rm{Q}} \right]$$

**Fig. 8:**
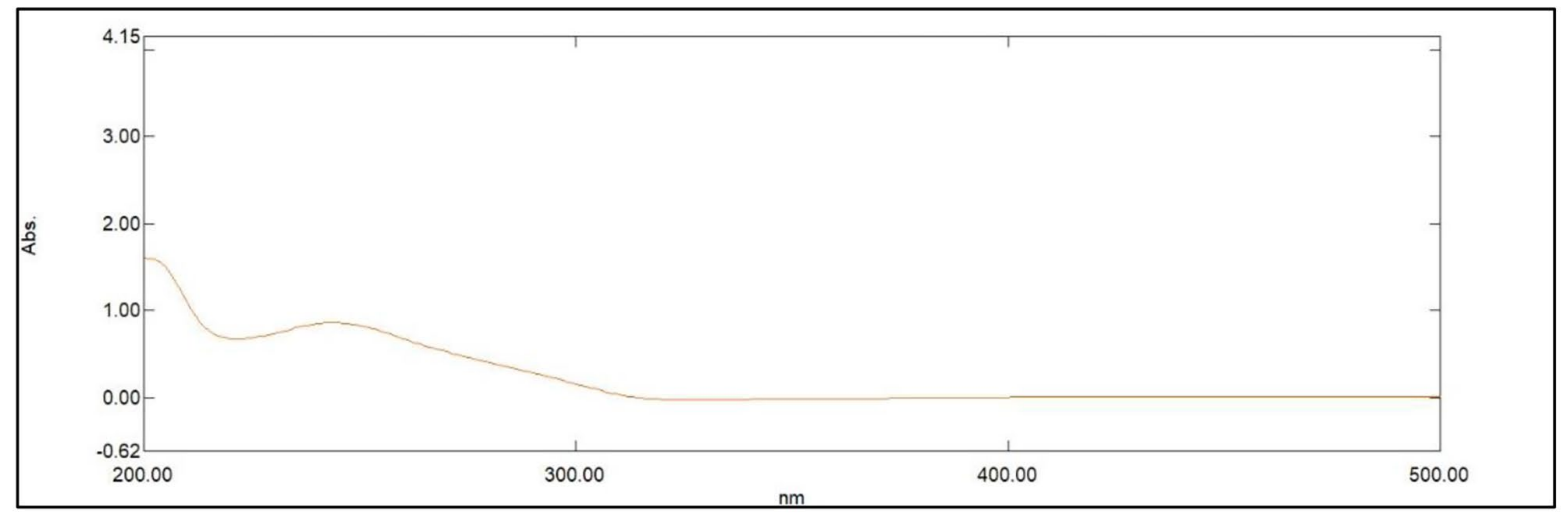
The UV–VIS absorption spectrum of FRS.

In this equation:

[Q] is the quencher molarity, F and F_0_ are the fluorescence intensities in presence and absence of quencher, respectively. τ ^0^ is the average lifetime of fluorescence (10^− 8^ s). K_sv_ and K_q_ represent the Stern-Volmer quenching constant and the quenching rate constant. The quenching experiments were done at ambient temperature. Quenching values were applied to the Stern Volmer`s equation resulting in Kq value of 3.96 × 10^12^ which was larger than (2.0 × 10^10^ L.mol^− 1^.s^− 1^) the maximum diffusion rate constant [23]. The K_q_ values at 303 k and 313 k were 3.31 × 10^12^ and 2.02 × 10^12^. Consequently, the mechanism is assumed to be static quenching. In this study, UV-VIS absorption spectrum of FRS overlapped with the excitation spectrum of NCQDs (Fig. 8). Thus, Inner Filter Effect (IFE) might take place. The correction of N-CQDs fluorescence intensity for possible IFE was studied upon adding increased concentrations of FRS, the corrected fluorescence intensity was expressed using Eq. (3) [24]:

3 $${{\rm{F}}_{{\rm{corr}}}} = {\rm{ }}{{\rm{F}}_{{\rm{obs}}}} \times {10^{\left( {{\rm{Aex + Aem}}} \right){\rm{/2}}}}$$

In which:

F_corr_ is the corrected fluorescence intensity after excluding inner filter effect from F_obs_, F_obs_ represents the observed fluorescence intensity, A_em_ and A_ex_ are the absorbance of the drug at the emission wavelength and excitation wavelength of N-CQDs. The suppressed efficiency (%E) was calculated for observed and corrected fluorescence intensities using Eq. 4:

4 $$\% {\rm{E }} = {\rm{ }}\left[ {1 - \left( {{\rm{F}}/{{\rm{F}}_0}} \right)} \right] \times 100$$

**Fig. 9:**
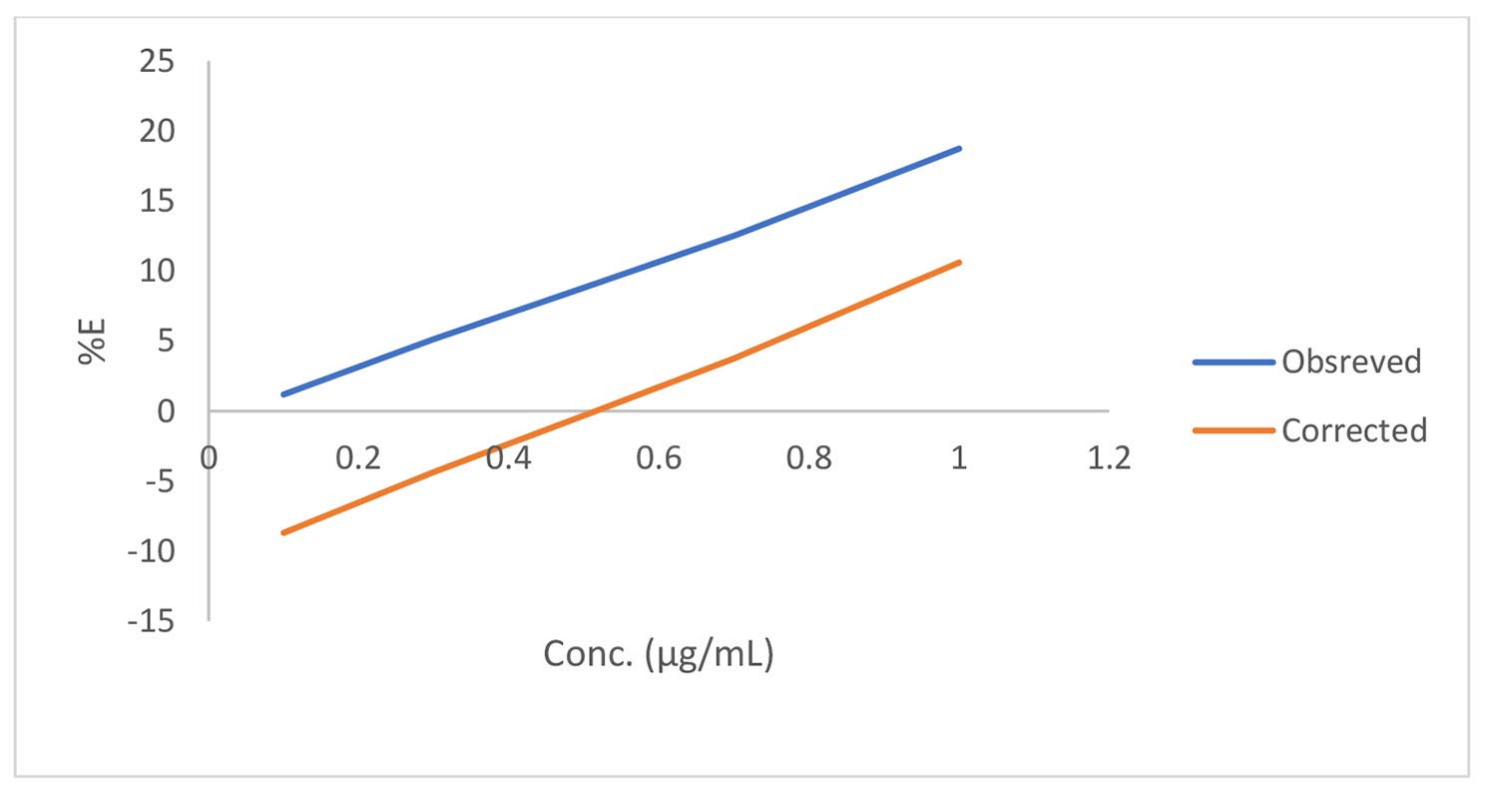
Plot of %E against concentrations of FRS.

%E of both corrected and observed fluorescence intensities of N-CQDs against drug concentration plot (Fig. 9) revealed that, IFE has a significant role in the quenching of N-CQDs fluorescence intensity by the cited drug.

### Method optimization and development

#### Effect of pH

The effect of pH on quenching of fluorescence intensity by FRS was studied using BRB solution over pH range from 2.1 to 12. Maximum ΔF value was obtained at pH 6.

### Effect of volume of the buffer

To investigate the effect of volume of buffer, increasing volumes of BRB were used. The maximum quenching effect on the fluorescence of N-CQDs was attained using 1 mL of BRB buffer of pH 6.

### Effect of volume of N-CQDs

Upon adding different volumes of N-CQDs, it was found that the optimum volume of N-CQDs is 400 µL. As it gave maximum fluorescence quenching with the drug.

### Effect of temperature

**Fig. 10:**
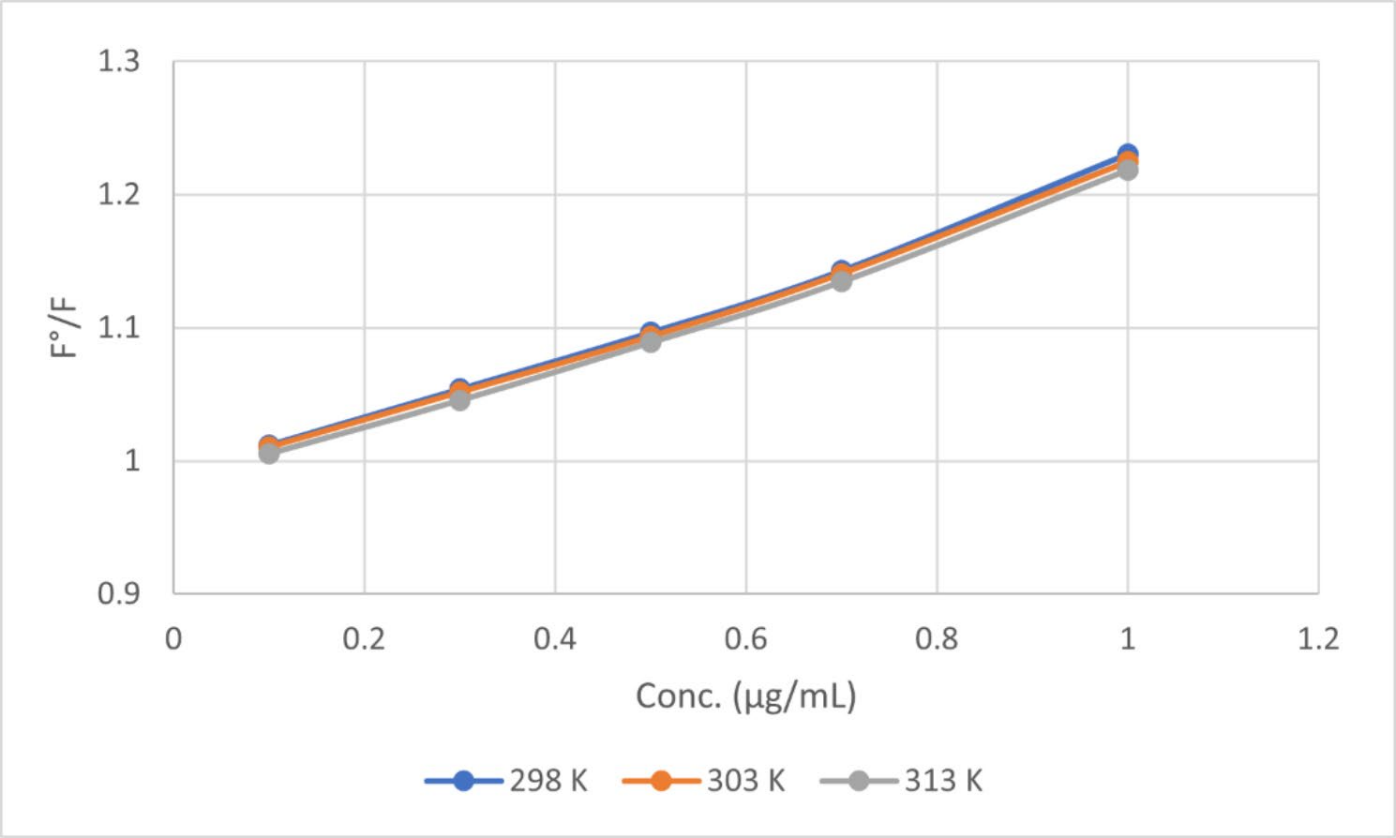
Stern Volmer plot of F°/F against different drug concentrations at different Temperatures

The influence of temperature on the relative fluorescence intensities was studied. The fluorescence intensities were recorded at 376 nm at 298, 303, 313 K. As temperature increased, the degree of quenching decreased (Fig. 10). As a result, the study was carried out at ambient temperature.

### Effect of incubation time

After adding the studied drug to N-CQDs, the fluorescence emission spectra at various time intervals were recorded starting from 1 to 40 min. The reaction was found to be instantaneous and 1 min was enough to complete the reaction. The fluorescence signals were stable for 30 min after which the quenching effect decreased significantly.

### Method validation

ICHQ2(R1) guidelines [25] were used to check the validity of the proposed method. The plot of concentrations in µg/mL of the drug against relative quenched fluorescence intensity was used to assess the linearity of the proposed method. The linear range was found to be 0.1 to 1.0 µg/mL. Equation 5 represents the linear regression:

5 $$\left( {{{\rm{F}}_0} - {\rm{F}}} \right)/{{\rm{F}}_0} = {\rm{ }}0.193{\rm{C }} - 0.0079\;\;\;\;\;\;\left( {{R^2} = 0.9994} \right)$$

where F is the fluorescence intensity of N-CQDs in the presence of studied drug, F_0_ is the fluorescence intensity of N-CQDs in the absence of FRS, C is the drug concentration in µg/mL.

The values of LOD and LOQ proved that the suggested method is sufficiently sensitive as summarized in Table 1 The accuracy of the proposed method was confirmed by calculating the mean % recoveries of raw material of FRS. The comparison method [4] depends on measuring the absorbance of FRS at 277 nm in methanol. Variance F-test and Student’s t-test were used to compare the results obtained from the comparison and proposed method as illustrated in (Table 2) [26]. There was no significant difference between the two methods regarding accuracy and precision.

**Table 1:** Analytical performance data for the proposed Method

Parameter	FRS
Linearity range (µg/mL)	0.1-1.0
Limit of detection (LOD)	0.029
Limit of quantitation (LOQ)	0.087
Correlation coefficient	0.9997
S.D. of residuals (S_y/x_)	0.0019
S.D. of intercept (S_a_)	0.0017
S.D. of slope (S_b_)	0.0028

**Table 2:** Assay results for the determination of the studied drug in raw material by the proposed and comparison method

	Proposed method			Comparison method [4]
	Concentration taken ^a^ (µg/mL)	Concentration found (µg/mL)	% Found	% Found
	0.10 0.30 0.50 0.70 1.0	0.102 0.306 0.496 0.687 1.009	101.96 101.97 99.17 98.17 100.91	100.73 99.27 100.24
X̄± SD			100.44 ± 1.7	100.08 ± 0.74
*t-*test			0.33 (2.44)^b^	
*F*-test			5.29 (19.25)^b^	

^a^ Each result was the average of three separate determinations ^b^ The figures between brackets were the tabulated *t* and *F* values at P = 0.05

The precision of the method was evaluated by investigating the inter-day and intraday precisions. The %RSD values were calculated using three replicates of three different concentrations in three successive days and in the same day. The developed method exhibited a relatively lower values of % RSD than 2% which is acceptable (Table 3). Changing the temperature by ± 2 C and the pH by ± 0.2 gave no change on the study results. Those minor and small changes may arise during everyday work. As a result, the robustness of the method was confirmed. The % recoveries of FRS in its dosage forms were calculated to ensure the selectivity of the proposed method as illustrated in (Table 4). From the obtained results, it was obvious that the common tablet excipients didn`t interfere with the results of the method. Thus, the method could be used for the estimation of FRS in its pharmaceutical preparations. The selectivity of the method was confirmed by studying the interference from some co-administered drugs, such as valsartan and spironolactone. The tolerance limit for valsartan was 1.0 µg/mL, and for spironolactone, it was 0.5 µg/ml.

**Table 3:** Intraday and inter-day precision data for the determination of the studied drug by the proposed method

Conc. taken in µg/mL	Intraday ^a^			Inter-day ^b^		
	Conc. found ± S.D. (µg/mL)	%RSD	%Error ^c^	Conc. found ± S.D. (µg/mL)	%RSD	%Error ^c^
0.3	0.3 ± 0.43	0.42	0.25	0.3 ± 0.54	0.53	0.31
0.5	0.5 ± 0.16	0.16	0.09	0.5 ± 0.91	0.91	0.53
0.7	0.7 ± 0.37	0.37	0.22	0.7 ± 1.07	1.08	0.62

Each result is the average of three separate determinations

^a^ Within the day

^b^ Three consecutive days. ^c^ % Error = % RSD/ √n

**Table 4:** Assay results for determination of FRS in its dosage forms by the proposed method

Preparation	Proposed method			Comparison methods [4]
	Amount taken^a^ (µg/mL)	Amount found (µg/mL)	% Recovery	
a-Lasix20 mg/2mLampoules	0.1	0.98	98.96	101.03
	0.2	0.202	101.04	98.97
	0.3	0.299	99.65	100.34
‾x			99.88	100.11
± SD			± 1.06	± 1.05
% RSD			1.06	
% Error			0.61	
t-test			0.27(2.77)^b^	
F-test			1.02(19)^b^	
b-Lasix® 40 mg tablets	0.1	0.102	102.08	102.90
	0.2	0.196	97.92	97.09
	0.3	0.302	100.69	100.96
X̅			100.23	100.31
± SD			± 2.12	± 2.96
% RSD			2.11	
% Error			1.22	
t-test			0.04(2.77)^a^	
F-test			1.95(19)^a^	

^a^ Each result was the average of three separate determinations

^b^ The figures between brackets were the tabulated *t* and *F* values at P = 0.05^26^

a- Ampoule contains20 mg/ 2 mL FRS. b-Tablets contain 40 mg FRS.

### Applications

#### Drug analysis in its Pharmaceutical Preparations

FRS was successfully estimated in its pharmaceutical preparations by the suggested method. The numerical results in (Table 4) show a good agreement and conformity with those obtained from the comparison method [4]. Variance ratio F-test and Student’s t-test [26] were used to statistically analyze the results obtained by both methods. No significant difference was observed between the calculated and tabulated values.

### Analysis of Spiked Human plasma

**Fig. 11:**
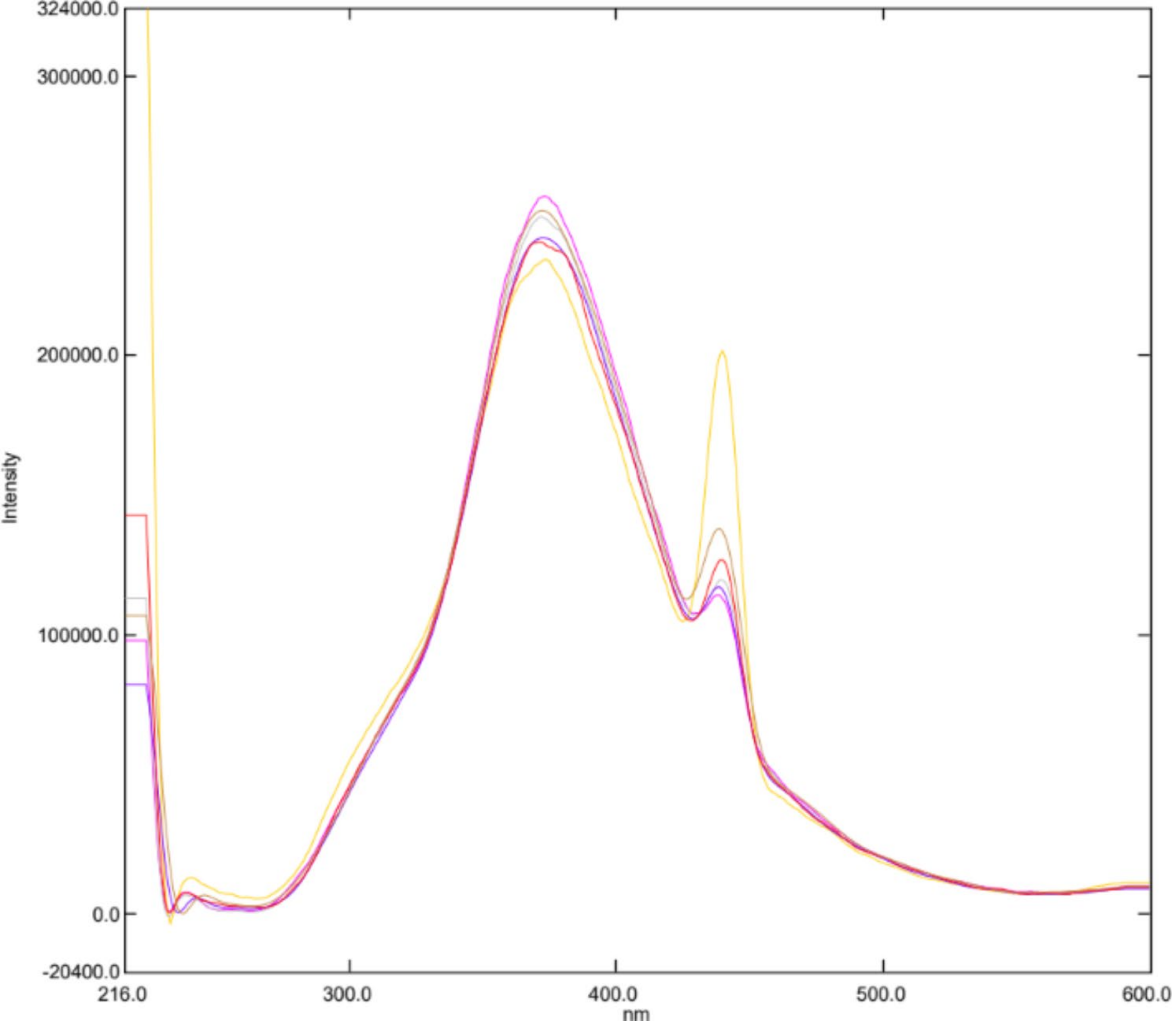
Fluorescence emission spectra of the N-CQDs under optimum conditions upon spiking of various concentrations of FRS to human plasma

After oral dosing of 40 mg FRS, the peak plasma concentration is 1.163 µg/mL [27], as a result the method could be used in the estimation of the drug in plasma. After preparing the samples, they were analyzed as described before. The method proved its suitability to estimate FRS in the studied matrix. The range of the average recovery values was from 89.16 to 114.33% as shown in Table 5 and Fig. 11.

**Table 5:** Application of the proposed spectrofluorimetric method for determination of FRS in spiked human plasma

Drug	Conc. added (µg/mL)	Conc. found (µg/mL)	%Recoveries
FRS	0.1	0.089	89.16
	0.2	0.179	89.84
	0.3	0.343	114.33
	0.4	0.403	100.82
	0.6	0.585	97.49

## Conclusion

Ecofriendly nano fluorescent sensors (N-CQDs) were successfully synthesized and applied to the estimation of FRS in pharmaceutical preparations as well as spiked human plasma. The water-soluble N–CQDs were prepared using sucrose and urea, the size of N-CQDs was distributed in the range of 6.63 to 10.23 nm with an average of 8.2 nm. They are highly stable and have strong fluorescence intensity. The proposed method is characterized by being highly sensitive, selective, simple and low cost.

## Data Availability

The datasets generated and/or analyzed during the current study are available from the corresponding author on reasonable request.

## Abbreviations

N-CQDS: Nitrogen doped carbon quantum dots
FRS: Furosemide
BRB: Britton Robinson Buffer
CQDS: Carbon quantum dots
IFE: Inner filter effect
